# Editorial: Applications of metabolomics to the discovery of biomolecules from natural products

**DOI:** 10.3389/fmolb.2023.1190730

**Published:** 2023-04-06

**Authors:** Young Hae Choi, Young Pyo Jang, Michel Frédérich

**Affiliations:** ^1^ Natural Products Laboratory, Institute of Biology, Leiden University, Leiden, Netherlands; ^2^ College of Pharmacy, Kyung Hee University, Seoul, Republic of Korea; ^3^ Pharmacognosy Laboratory, Center for Interdisciplinary Research on Medicines—CIRM, Université de Liège, Liège, Belgium

**Keywords:** metabolomics, methodologies, bioactivity, natural product-originated drugs, identification

Natural products are an undoubtedly reliable and robust source of biomolecules for pharmaceuticals, cosmetics, and nutraceuticals. Their value as a chemical platform lies greatly in the range of structural diversity they offer. This diversity, while an advantage, implies that to fully exploit the bounties of this source, the experimental design must consider the variability of potentially interesting compounds and the fact that they are often immersed in complex chemical environments. Furthermore, revealing the activity and mechanism of action of new biomolecules can be challenging due to the vast spectrum of biosystems involved. Therefore, the key Research Topic in this field is how to handle the complexity of these chemical and biological networks in organisms and use them to our advantage. Metabolomics, with its holistic and comprehensive approach, is well-suited for this purpose.

In recent years, systems biology has become a widely accepted paradigm in life sciences research. The data generated from metabolomics provides valuable information which integrated with all other “omics” data allows the investigation of biological processes from this holistic approach. The goal of metabolomics is to work out a biological or physiological phenomenon based on data obtained from chemical profiling of the metabolome. In recent decades, the impressive technological advances in the analytical platforms required for metabolomics have allowed increased sensitivity, data robustness and resolution, which together with new sample preparation techniques and the development of data processing and mining methods have widened the range and scope of the field resulting in successful nature-based drug development and the investigation of their mechanisms.

In this context, the global aim of this Research Topic is to cover all metabolomics-based applications and methods directed at the discovery of useful biomolecules from natural sources, and the mechanisms of their bioactivity. For this, we have selected these eight articles that represent current research trends of the application of metabolomics:

- Methodological development of metabolomics techniques
- Applications of metabolomics within bioactivity tests
- Development of metabolomics-based bioscreening methods
- Investigation of the efficacy of natural product-originated drugs
- Identification of bioactive molecules from natural products based on metabolomics techniques

The aim of a metabolomic experiment is to detect and identify all the metabolites that exist within an organism. This premise requires the extraction of the whole metabolome from the sample, as, simply speaking “what you see is what you extract.” Thus, a number of extraction methods have been developed for various organisms including plants, microbes, mammalians, and humans. The optimization of these methods involves not only tailoring them to each organism but to the specific tissues in a given organism. Andersen et al. reported a comprehensive study comparing ten extraction protocols in four human tissues (liver tissue, bone marrow, HL60, and HEK cells) using 630 metabolites of different classes. Based on their results the authors concluded that the coverage of extracted metabolites is solvent-dependent, having implications in the design of the measurements of the sample types and metabolites of interest.

The chemical diversity of the metabolome, while its major asset, leads to the need of time and cost-consuming steps to detect a bioactive chemical with the risk of losing promising metabolites along the way. The work of Bonnet et al. is a good example of how to apply metabolomics to find specific metabolites for a disease model. Using HPLC-ESI(+)-Q/TOF, 12 Strychnos extracts with antiplasmodial activity were chemically profiled and analyzed using molecular networking, yielding a promising indole alkaloid with antiplasmodial activity.

Recently emerged OMICS technologies have been used to prove the existence of interactions between organisms, for example, the interplay between plants and microbes *via* endophytes. Endophytes have proved to be responsible for the synthesis of bioactive molecules in their host plant. Using MS-based metabolomics, Lee et al. discovered that the endophyte strain *Salinivibrio costicola* isolated from *Suaeda maritima* (L.) Dumort allowed the accumulation of polyhydroxybutyrate.

The development of new medicinal drugs generally requires their *in-vivo* testing to evaluate their efficacy in humans. This generally involves multiple reactions in an *in-vivo* model, and their untargeted analysis. The work of Wang and Sun is a good example of the application of metabolomics to understand a pharmacological mechanism. The MS-based chemical profiling of serum from rats fed with the diterpene, daturoside, which has anti-inflammatory activity against acute gouty arthritis, showed that the activity could be linked to the metabolism of amino acids, sugar, fatty acid, energy, purine and butanoate. Using a similar approach, Park et al. applied liquid chromatography-based chemical profiling to reveal the bioactivity of ginseng fermented with golden flower fungus. The biotransformed ginseng showed potent anti-aging efficacy on the skin attributable to the ginseng metabolites.

Agricultural products have also been targets of metabolomics. Witte et al. used MS-based metabolomics to identify mycotoxins of *Alternaria* section *Alternaria*, prolific producers of metabolites known to act as virulence factors of disease. Their data showed that three of 36 strains produced the dehyrocurvularian family of toxins and associated detoxification products.

Using a popular approach in the metabolomics field, Fernandes et al. identified *Citrus* components that were active against *Citrus* black spot disease, responsible for enormous losses of *Citrus* production. Comparing a susceptible *Citrus* species and a resistant *Citrus* species, they identified various prenylated coumarins with possible activity against the pathogenic fungus.

Finally, Quiros-Guerrero et al. report a systematic approach for the search of new molecules from natural product extracts based on the computational tool, *Inventa*. In the article, the authors introduce the tool that reveals structural novelty potential within extracts using untargeted mass spectrometry data, spectral annotation, and literature reports. This is illustrated by its application to a set of plants from the Celastraceae family, resulting in the isolation and *de novo* characterization of thirteen new dihydro-*β*-agarofuran sesquiterpenes, five of which presenting a new 9-oxodihydro-*β*-agarofuran base scaffold.

We are very pleased to be able to bring this interesting range of research papers together in this edition and thank the authors for their valuable contributions with the hope that these articles will be a guide for future research in the field.

